# Supplementary material for: WordCommentsAnalyzer: A windows software tool for qualitative research
Source: F1000Res. 2018 Sep 4;7:536. Originally published 2018 May 3. [Version 2] doi: 10.12688/f1000research.14819.2 (PMC6137411; doi:10.12688/f1000research.14819.2)
Supplement: Supplementary file 3 [file f1000research-7-17614-s0002.tgz › b2a79496-4ada-47c4-9682-a99ce867836d.zip!/Eight Iranian Health Journals/Archives of Rehabilitation_68.docx]

© Archives of Rehabilitation. This work is licensed under CC BY-NC 4.0 (https://creativecommons.org/licenses/by-nc/4.0/).

Volume & Issue: دوره 3 - شماره 1 و 2

Title: بررسی وضعیت انواع فلج و قطع اندام در ایران

http://rehabilitationj.uswr.ac.ir/article-1-554-fa.html

هدف: این مقاله حاصل یک بررسی مقطعی از وضعیت برخی معلولیت ها (انواع فلج و قطع اندام) در ایران به منظور برآورد شیوع و شناخت علل آن ها است. روش بررسی:‌ روش نمونه گیری سیستماتیک و خوشه ای و اندازه هر خوشه، 15خانوار در نظر گرفته شده است. نسبت نمونه گیری یک هزارم جمعیت کشور در نظر گرفته شده و در مجموع، 59678 نفر مورد بررسی قرار گرفته اند. یافته‌ها: معلولیت های (فلج و قطع اندام)، شیوعی برابر 7/0 در هزار دارند که از بین آن ها، بیش ترین شیوع با رقم 2/8 در هزار نفر مربوط به فلج از نوع همی پلژی و پاراپلژی است. شیوع فلج مغزی 2/0 در هزار، قطع اندام ها 1/3 درهزار و فلج اطفال 0/9 در هزار نفر است. با انجام واکسیناسیون وسیع در سراسر کشور که بعد از جمع آوری اطلاعات این بررسی انجام شد، گزارش های کنونی حاکی است که فلج اصفال در سنین پایین، شیوع بسیار ناچیزی دارد.24/5  درصد از علل مربوط به انواع فلج و قطع اندام «مادرزادی» است، 25/5 درصد ناشی از «آسیب فیزیکی»، 15/6 درصد حاصل بیماری های عفونی، 28/8 درصد از «بیماری های غیر عفونی» و 3/1 درصد پیامد «کهولت و پیری» بوده اند. 67/2 درصد از علل، مربوط به فلج مغزی "مادرزادی" است. 54/8 درصد فلج های همی پلژی و پاراپلژی ناشی از «بیماری های غیر عفونی» است. هم چنین 73/7 درصد قطع اندام ها به علت "آسیب فیزیکی" است. با فزایش سن، شیوع فلج های پاراپلژی ، همی پلژی و قطع اندام، به طور چشم گیری افزایش می یابد که بیش ترین شیوع با رقم 29/0 در هزار، به افراد 65 سال و بزرگ تر تعلق دارد. شیوع معلولیت های فوق در مردان، 6/3 در هزار و در زنان، 2/8 در هزار است که به طور نسبی، در مردان کمی بیش از دو برابر زنان است (P<0/000). نتیجه‌گیری: به طور کلی، شیوع این معلولیت ها در مردان بیش تر از زنان است. در مجموع، شیوع همی پلژی، پاراپلژی و قطع اندام در مناطق شهری، 4/9 در هزار و در مناطق روستایی، 4/1 در هزار نفر است که در حضور متغیر سن، اختلاف معنی داری بین مناطق شهری و روستائی وجود ندارد (P<0/059). استفاده از مدل رگرسیون لجستیک نشان داد که هریک اسه مبغیر سن، جنس و محل سکونت، به تنهایی بر شیوع مجموع همی پلژی، پاراپلژی و قطع اندام اثر معنی دار دارند (P<0/039). با منظور کردن دوبه دوی متغیرهای فوق در مدل، ملاحظه می شود که محل سکونت اثر خود را از طریق سن اعمال می کند، ولی هریک از دو متغیر سن و جنس، در حضور یکی دیگر از متغیرهای فوق، بر شیوع این معلولیت ها اثر معنی دار دارند. با منظور کردن هرسه متغیر به طور هم زمان در مدل، نتایج، نظیر حالت دو متغیر فوق است (P<0/000).

Title: ارزیابی سیر پیشرفت ناتوانی‌های عملکردی بیماران مبتلا به آمیوتروفیک لترال اسکلروز

http://rehabilitationj.uswr.ac.ir/article-1-555-fa.html

هدف: آمیوتروفیک لترال اسکلروز یک بیماری دژ نراتیو، پیش رونده و کشنده نورون حرکتی است. در این مقاله سیر پیشرفت ناتوانی های عملکردی بیمار و فاکتورهای موثر بر آن مورد بررسی قرار می گیرد. روش بررسی: پس از انتخاب 59 بیمارALS (براساس معیارهایEL.Escorial) برای هر بیمار در ابتدا و انتهای یک دوره پیگیری شش ماهه ALSFRS تعیین شد. از 59 بیمار، 9 بیمار طی دوره پیگیری فوت شدند. نسبت مرد به زن برابر با 1/9 بود. این نسبت در نوع بولبار0/8 و در نوع غیر بولبار 2/5 بود. شایع ترین گروه سنی مبتلا، گروه 65-40 ساله بودند (68%). یافته‌ها: شایع ترین لوکالیزاسیون شروع بیماری، دست‌ها بود. (46%) میانگین سنی بیماران، 53/4 سال بود. (گروه بولبار 59/1 سال و گروه غیر بولبار 51/7 سال) گروه سنی یا جنس بیمار در میزان پیشرفت ناتوانی‌ها تاثیر معنی داری نشان نداد، ولی لوکالیزاسیون بولبار سیر سریع تری نسبت به غیربولبار نشان داد. در نوع بولبار، زنان پیش از مردان مبتلا شدند (0/8=m/F) در بیماران فوت شده، میانگین مدت زنده ماندن24 ماه، در گروه بولباری 14 ماه و غیر بولباری 27 ماه بود. نتیجه‌گیری: گروه سنی بالاتر از 53 سال نسبت به گروه سنی  جوان‌تر، مدت بقای کمتری نشان داد. مدت بقا با جنس بیمار ارتباط معنی داری نشان نداد.

Title: مقایسه رفتار تطببیقی افراد عادی با افراد کم توان ذهنی

http://rehabilitationj.uswr.ac.ir/article-1-556-fa.html

هدف:‌ مقاله حاضر به منظور بررسی و مقایسه رفتارهای انطباقی در افراد عادی و افراد کم توان ذهنی روی 320 آزمودنی (246 نفر عادی، 74 نفر کم توان ذهنی) با دامنه سنی 18-7 سال انجام گرفت.  روش بررسی:‌ ابزار پژوهش مقیاس رفتار انطباقی، ویرایش تجدید نظر شده ی نسخه ی 1993 مقیاس رفتار انطباقی است که از مقیاس های رفتار انطباقی انجمن کم توانی ذهنی آمریکا در سال های 1969 و 1974 تهیه شده و شامل دو بخش که دارای 18 حوزه رفتار انطباقی است. داده های پژوهش با استفاده از برنامه ی آماری SPSS مورد تجزیه و تحلیل قرار گرفت. یافته‌ها: یافته‌ها حاکی از این است که افراد کم توان ذهنی نسبت به افراد عادی در حوزه های رفتار انطباقی: عملکرد مستقل، فعالیت اقتصادی؛ رشد زبان، اعداد و زمان، فعالیت شغلی–پیش شغلی، خودفرمانی، مسئولیت پذیری، رفتار مختل کننده ی فردی، فعالیت خانگی، اجتماعی شدن، رفتار اجتماعی، همنوایی کردن و قابل اعتماد بودن عملکرد پایین تری دارند.  نتیجه‌گیری:‌ در حوزه های رشد جسمانی، رفتار کلیشه ای و بیش فعالی، رفتار جنسی، رفتار خودآزاری و اشتغال اجتماعی، تفاوت معنی داری بین دو گروه مشاهده نشده. علل احتمالی این تفاوت ها و تشابه ها به اختصار مورد بحث قرارگرفته است.

Title: اپیدمیولوژی و اتیولوژی صدمات ارتوپدی ناشی از کار

http://rehabilitationj.uswr.ac.ir/article-1-557-fa.html

هدف: حوادث ناشی از کار به دلیل هدف قرار دادن جمعیت جوان و فعال جامعه یکی از مهمترین گروه حوادث را تشکیل می دهند. هرساله تعداد زیادی از کارگران کشور بر اثر این نوع حوادث، سلامتی و کارایی خود را از دست می دهند. بررسی حاضر به صورت پیش نگر، با هدف شناسایی عمده ترین علل بروز این حوادث و دستیابی به راه های احتمالی جلوگیری از آن، در بیمارستان فیاض بخش انجام گرفت که یکی از فعال ترین بیمارستانهای کشور از نظر تعداد مراجعان سوانح حرفه ای می باشد. روش بررسی:‌ طی مدت یک ماه، بیمارانی که با صدمات ناشی از کار به بیمارستان مراجعه می کردند، به صورت متوالی وارد مطالعه شدند. 302 بیمار در این فاصله مورد بررسی قرار گرفتند. یافته‌ها:‌ صدمات دست بیشترین تعداد و شدیدترین نوع آسیب را که خود اختصاص داده بودند. نتیجه‌گیری:‌ مهم ترین عوامل قابل بررسی در بروز حوادث ناشی از کار عبارت بودند: از مشکلات روانی، سابقه بیماری، و عدم آموزش مناسب کارگران، وجود مشکلات مالی و عدم استفاده از وسایل ایمنی در بین عوامل بروز حادثه، میزان کمتری داشت.

Title: استفاده از بریس شست و توانبخشی زودرس در بیماران مشکوک به شکستگی اسکافوئید

http://rehabilitationj.uswr.ac.ir/article-1-559-fa.html

هدف: هر پزشکی در دوران طبابت خود به مرتب با بیماران مشکوک به شکستگی اسکافوئید برخورد می کند. در دو دهه ی اخیر تلاش های زیادی برای اصلاح برخورد کلاسیک با این عارضه که منجر به محرومیت بی مورد از کار تعداد زیادی از بیماران می شود، به عمل آمده است. در بیمارستان دکتر شریعتی و شهید معیری تهران در ظرف 8 ماه، مطالعه ای آینده نگر بر روی 51 بیمار با تشخیص اولیه ی شکستگی مشکوک اسکافوئید به عمل آمد. روش بررسی: درمان به صورت بی حرکتی با آتل شست و معایه ی بالینی هفتگی انجام گرفت   یافته‌ها: از نتایج حاصله مشخص شد که %10 موارد بیماران دارای ضایعه ای، نیازمند به بی حرکتی هستند. در %90 موارد، بهبودی حداکثر ظرف سه هفته حاصل شد که بیش از نیمی از آنان مدت نیاز به بی حرکتی به کم تر از دو هفته تقلیل یافت. نتیجه‌گیری: لذا به نظر می رسد تا هنگامی که روش غربالگری مناسب تری از معاینه ی بالینی در دسترس نباشد، بی حرکت کردن با بریس شست و معاینه ی هفتگی و توان بخشی زودرس روش مناسبی برای جایگزینی روش معمول درمان این عارضه یعنی گچ اسپایکای شست و معاینه بالینی بعد از دو تا سه هفته می باشد.

Title: معرفی ۶۰ مورد اسپاسم شیر خواران ار نطر اتیولوژی علائم بالینی، EEG، Brain CT Scan در بیمارستان مقید در سال‌های 79-77

http://rehabilitationj.uswr.ac.ir/article-1-560-fa.html

هدف: در بین انواع مختلف سندرم های اپی لپتیک، اسپاسم شیرخواران یکی از بدخیم ترین آنها تلقی می شود، چرا که اثرات سوء ترمیم ناپذیری بر مغز کودک اعمال می کند و هر قدر طول مدت اسپاسم شیرخواران بیشتر باشد نتایج مخرب آن هم وخیم تر است. اکثر مبتلایان به این بیماری، کودکان کمتر از یکسال هستند و فقط 5 درصد موارد آن در سنین بالاتر از یکسال تظاهر می کند. هدف این پژوهش مطالعه کودکان 24-2 ماهه مبتلا به اسپاسم شیرخواران است. روش بررسی: این مطالعه  توصیفی بر روی 60 کودک 2-24 ماهه مبتلا به اسپاسم شیرخواران که بین سالهای 79-1377 به دپارتمان اعصاب کودکان بیمارستان مفید مراجعه کرده بودند، با تکنیک مشاهده، مصاحبه، معاینه و استفاده از پرسشنامه انجام گرفت. بیماران مورد بررسی 36 پسر و 24 دختر بودند. یافته‌ها: از نظر اتیولوژی، 48 مورد (%80) سمپتوماتیک و 12 مورد (%20) کریپتوژیک و ایدیوپاتیک بودند. از نظر تظاهرات بالینی، 35 مورد (%58) از نوع فلکسور، 6 مورد (%10) از نوع اکستنسور و 19 مورد (%22) از نوع مخلوط بودند. در الکتروانسفالوگرافی به عمل آمده از بیماران، در همه موارد هیپس آریتمی دیده شد و سی تی اسکن مغزی بیماران در 11 مورد (%20) آتروفی مغزی را نشان داد و بقیه موارد (%80) نرمال بود. نتیجه‌گیری: در مطالعه ما نوع سمپتوماتیک و فلکسور شایعتر بود. هیس آریتمی در الکتروآنسفالوگرافی همه بیماران دیده شد و سی تی اسکن مغزی در بیشتر موارد نرمال بود.

Title: مقایسه فراوانی علائم ظریف عصبی در اختلالات روان پزشکی

http://rehabilitationj.uswr.ac.ir/article-1-561-fa.html

هدف: وجود علایم ظریف عصبی در اختلالات روانی همواره مدنظر صاحب نظران بوده است. هدف از این پژوهش آن بود که با توجه به کاربردی بودن این علایم در مواقعی که شک تشخیصی وجود دارد ، یک بار دیگر این رابطه با توجه به اطلاعات جدید در بیماران مختلف روان پزشکی در بیمارستان شهید بهشتی کرمان مورد بررسی قرار بگیرد و فراوانی آن در بیماران مختلف با یکدیگر مقایسه شود. روش بررسی: بر این اساس، 213 بیمار که دارای تشخیص نهایی اختلال خلقی – اختلال اضطرابی و اسکیزوفرنی بودن بطور متوالی انتخاب شدند. این حجم نمونه بر اساس 93% اطمینان و نسبت %32 و دقت %6 از یک مطالعه مقدماتی بدست آمد. شش آزمایش علائم ظریف عصبی بر روی بیماران انجام گرفت. یافته‌ها: نتایج حاکی از آن است که رابطه سن با 4 مورد از تست‌ها، یعنی Standing on one foot Heel Walking Toe walking Tandem walk معنی‌دار است و رابطه‌ی اختلالات روانی با دو مورد از تست ها معنی دار بود که عبارت بودند از تبعیض دو سویه (PV=0/01) و اکستروژن زبان (PV=0/02) که اولی در افسردگی و دومی در اسمکیزوفرنی بالاتر بود و در باقی موارد رابطه ی معنی داری وجود نداشت. نتیجه‌گیری: حاصل این پژوهش نشان دهنده ی آن بود که فراوانی اکثر N.S.Sها در اختلالات روانی برابر است و اختلاف اندکی با یکدیگر دارند.

Title: خلاصه‌ای از تحقیقات انجام‌شده روی سیستم ایمنی سلولی سالمندان با استفاده از آزمون‌های تکثیر سلولی

http://rehabilitationj.uswr.ac.ir/article-1-562-fa.html

یک از اولین تظاهرات مشهص شده همراه با سن تغییر در کاهش پاسخ به واکنش DTHاست. پاسخ DTH یک مثال بارز از پاسخ سلولهای Th است که توسط ترشح سیتوکائین های تیپ 1-TH به وجود می آید. اگر چه اتفاقات و رخدادهای زیادی قبل از تکثیر سلولی می بایست به وجود بیاید تا امر تکثیر صورت گیرد، ولی چون تکثیر سلولی به عنوان یک عامل مشخص کننده در افراد مورد استفاده قرار می گیرد، بنابر این بسیاری از تحقیقات، پاسخهای تکثیری را به عنوان بررسی تغییرات همراه با سن در پاسخ سلولهای T مورد ارزیابی قرار می دهند. کاهش توانمندی تکثیر سلولهای PBMC در پاسخ به تحریک سلولهای T، بیشترین میزان تغییرات همراه با سن را در سیستم ایمنی نشان می دهد. گزارش شده است که %40 از افراد بالای 70سال که وضعیت بهداشتی و اجتماعی مناسبی داشتند، در آزمایش پوستی کاهش واکنش DTH را نشان می دهند. یکی از دلایل کاهش در پاسخ DTH می تواند در اثر تغییر در فعال شدن اولیه سلولهای T باشد، به عبارتی، تغییر در تولید سیتوکائین ها توسط سلولهای فعال شده یا فعالیت تغییر یافته توسط ماکروفاژها باشد. همچنین تغییرات همراه با سن به دفعات زیادی در In vitro (پاسخ به آنتی ژنهایی همچون: دی تیتروکلروبنزن، استرپتوکیناز یا استرپتودورناز) مشاهده شده است که شامل، کاهش در پاسخ های تکثیر سلولی، تغییر در پیامهای فعال کنندگی و تغییر در تولید سیتوکائین ها است. بعلاوه، تحقیقات مهمی در دو دهه گذشته روی تغییرات ظاهری ایجاد شده بر روی سلولهای T همراه با افزایش سن انجام گرفته و به طور عمومی پذیرفته شده است که سالمندی با تغییرات زیادی در واکنش های ایمنی همراه است و این امر به صورت تغییرات بسیار بارز در مجموعه سلولهای T قابل مشاهده است. بنابراین، این تغییرات باید مراقبان بهداشت را وادار کند توجه خاصی به سالمندی داشته باشند.

Title: بررسی اثر تکنیک‌های کرانیوساکرال در درمان سردردهای مزمن

http://rehabilitationj.uswr.ac.ir/article-1-563-fa.html

در این تحقیق اثر درمانی تکنیکهای کرانیوساکرال بروی خانم 47 ساله‌ای با سابقه سردرد شدید به مدت 5 سال مورد بررسی قرار گرفت. سردرد بیمار بطور مداوم در طول روز و شدیدتر در صبح و شب از پشت سر شروع می شد و به گوش، شقیقه فک راست و گاهی تمام سر انتشار می یافت.هم چنین ماهی یک بار 2 تا 3 روز قبل از قاعدگی شدت سردرد به حداکثر خود می رسید. علاوه بر سردرد بیمار از وزوز گوش و سرگیجه شکایت داشت طبق بررسی های مختلف علتی برای سردرد او بیان نشده بود. انجام درمانهای مختلف، از جمله یک دوره فیزیوتراپی جهت اصلاح دیسفانکشنهای ستون فقرات تاثیر در بهبودی سردرد بیمار نداشت این بررسی بصورت A-B-A Single Subject Design انجام گرفت. که شامل یک هفته دوره قبل از درمان، 4 هفته دوره درمانی به صورت هفته ای یک جلسه و یک سال دوره پیگرد درمانی بود. فاکتورهای موردسنجش شدت درد، تداخل درد در خواب و فعالیت عمومی بودند، که میزان درد بر اساس visual analog scale از 7/5 در قبل از درمان به 3 پس از دوره درمانی و 1 بعد از یک سال پیگرددرمانی رسید. تداخل درد درخواب از %50 در دوره قبل از درمان به %30 پس از دوره درمان و 0% بعد از درمان رسید. تداخل درد در فعالیت عمومی از %80 دردوره قبل از درمان به %40 پس از دوره درمانی و %0 بعد از یکسال رسید. وزوزگوش و سرگیجه بیمار بطور کلی برطرف شد و فقط سردردی که 2 تا 3 روز قبل از قاعدگی بروز می گردد با شدت و وسعت بسیار کمتری باقی مانده بود.

Title: نورپاتی ناشی از گیرافتادن شاخه داخلی عصب سورال

http://rehabilitationj.uswr.ac.ir/article-1-564-fa.html

دردهای مزمن پشت ساق علل مختلف دارد که شایع ترین آنها شکستگی استرس، سیندروم کمپارتمنت مزمن، عضله سولئوس فرعی، و کشیدگی های عضلانی می باشند. یکی از علل دردهای مزمن ساق، گیرافتادن اعصاب یا نوروپاتی انترپمنت در اعصاب ساق پا است که چون یافته غیر شایعی است در اغلب موارد تشخیص داده نمی شود و یا تاخیر تشخیص داده می شوند. بیمار ما آقای 15 ساله ای بود که به دلیل در مزمن ساق مدت ها تحت درمان های مختلف قرار گرفته، ولی درد وی همچنان ادامه داشت.  کلیه بررسی های پاراکلینیکی و الکترودیاگنوستیک نرمان گزارش شده بود. پس از آزاد کردن آپونوروز عصب سورال با جراحی کلیه علائم بیمار رفع شد. نوروپاتی ناشی از گیرافتادن عصب سورال در نواحی مختلف از ساق و یا مچ پا و یا پا می تواند رخ دهد اما درگیری عصب در ناحیه خلف ساق به عنوان یکی از علل درد مزمن ساق به خصوص در ورزشکاران بیان شده است. ما در بررسی مقالات گزارش مبنی بر گیر افتادن شاخه داخلی عصب سورال در محل خروج از فاسیای کرورال در افراد عادی پیدا نکردیم. در این بیماران تشخیص تنها بر اساس تاریخچه و معاینه بالینی مسیر است و در اغلب موارد تست های الکترود یا کنوستیک نرمال می‌باشد.

Title: بررسی موارد فلج عصب سیاتیک به‌دنبال ترزیق آمپول دیکلوفناک سدیم در ناحیه سرینی و نتایج عمل جراحی در آنها

http://rehabilitationj.uswr.ac.ir/article-1-565-fa.html

یک از علل شایع ضایعه عصب سیاتیک در شیرخواران و کودکان، تزریق عضلانی داروها به ناحیه سرینی است که دو عامل مهم در ایجاد آن موثر است -1 نوع ماده تزریق -2 محل تزریق  در این مقاله، سی و دو مورد آسیب عصب سیاتیک به دنبال تزریق آمپول دیکلوفنات سدیم بررسی شده که 8 مورد از این بیماران تحت عمل جراحی قرار گرفته اند و در همه موارد، نتیجه رضایت بخش بوده است، در ضمن موارد گزارش شده مربوط به فروردین  1378تا آذر ماه 1377 است.
